# Diagnostic utility of zinc protoporphyrin to detect iron deficiency in Kenyan pregnant women

**DOI:** 10.1186/s12916-014-0229-8

**Published:** 2014-11-26

**Authors:** Martin N Mwangi, Sumi Maskey, Pauline EA Andang’o, Noel K Shinali, Johanna M Roth, Laura Trijsburg, Alice M Mwangi, Han Zuilhof, Barend van Lagen, Huub FJ Savelkoul, Ayşe Y Demir, Hans Verhoef

**Affiliations:** Maseno University, School of Public Health and Community Development, Private Bag, Maseno, Kenya; Wageningen University, Cell Biology and Immunology Group, P.O. Box 338, 6700 AH Wageningen, The Netherlands; Department of Food Technology and Nutrition, University of Nairobi, College of Agriculture and Veterinary Sciences, Applied Nutrition Programme, P.O. Box 442, Uthiru, Nairobi Kenya; Wageningen University, Laboratory for Organic Chemistry, Dreijenplein 8, 6703 HB Wageningen, The Netherlands; Meander Medical Centre, Laboratory for Clinical Chemistry, Maatweg 3, 3813 TZ Amersfoort, The Netherlands; MRC International Nutrition Group, London School of Hygiene and Tropical Medicine, London, WC1E 7HT United Kingdom; MRC Keneba, Private Bag, The Gambia London, UK

**Keywords:** Erythrocyte protoporphyrin, Iron deficiency, Kenya, Malaria, *Plasmodium*, Pregnancy, Zinc protoporphyrin

## Abstract

**Background:**

Iron-deficient erythropoiesis results in excess formation of zinc protoporphyrin (ZPP), which can be measured instantly and at low assay cost using portable haematofluorometers. ZPP is used as a screening marker of iron deficiency in individual pregnant women and children, but also to assess population iron status in combination with haemoglobin concentration. We examined associations between ZPP and disorders that are common in Africa. In addition, we assessed the diagnostic utility of ZPP (measured in whole blood and erythrocytes), alone or in combination with haemoglobin concentration, in detecting iron deficiency (plasma ferritin concentration <15 μg/L).

**Methods:**

Single blood samples were collected from a population sample of 470 rural Kenyan women with singleton pregnancies, gestational age 13 to 23 weeks, and haemoglobin concentration ≥90 g/L. We used linear regression analysis to assess associations between ZPP and iron markers (including anaemia), factors known or suspected to be associated with iron status, inflammation markers (plasma concentrations of C-reactive protein and *α*_1_-acid glycoprotein), infections (*Plasmodium* infection, HIV infection), and other disorders (*α*^+^-thalassaemia, plasma concentrations of total bilirubin, and lactate dehydrogenase). Subsequently, in those without inflammation, *Plasmodium* infection, or HIV infection, we used logistic discriminant analysis and examined receiver operating characteristics curves with corresponding area-under-the-curve to assess diagnostic performance of ZPP, alone and in combination with haemoglobin concentration.

**Results:**

Individually, whole blood ZPP, erythrocyte ZPP, and erythrocyte protoporphyrin had limited ability to discriminate between women with and without iron deficiency. Combining each of these markers with haemoglobin concentration had no additional diagnostic value. Conventional cut off points for whole blood ZPP (>70 μmol/mol haem) resulted in gross overestimates of the prevalence of iron deficiency.

**Conclusions:**

Erythrocyte ZPP has limited value to rule out iron deficiency when used for screening in conditions with a low prevalence (e.g., 10%). ZPP is of unreliable diagnostic utility when discriminating between pregnant women with and without iron deficiency. Based on these findings, guidelines on the use of ZPP to assess iron status in individuals or populations of pregnant women need review.

**Trial registration:**

NCT01308112 (2 March 2011).

## Background

Zinc protoporphyrin (ZPP) indicates the systemic supply of iron to erythrocytes in bone marrow. Iron-deficient erythropoiesis results in excess formation of ZPP, which can be measured instantly and at low assay cost using portable haematofluorometers. ZPP has been used as a screening marker to manage iron deficiency in children and pregnant women [1,2], with the reported advantage that values remain stable throughout gestation in women with adequate iron nutrition [3-5]. ZPP has also been recommended to be used in combination with haemoglobin concentration in surveys to assess population iron status [1,6]. We have concerns, however, about gross discrepancies between reported prevalence estimates for iron deficiency obtained by ZPP and circulating ferritin concentrations [7,8].

ZPP is the predominant form of non-haem protoporphyrin in normal erythrocytes [2]. Non-haem protoporphyrin also includes free erythrocyte protoporphyrin (FEP, i.e., the metal-free form that occurs naturally in erythrocytes). If the supply of iron is insufficient or when iron utilization is impaired (e.g., anaemia of chronic disease), zinc is used in the biosynthetic pathway of haem instead of iron, resulting in iron-zinc substrate competition for ferrochelatase and the formation of ZPP. Thus, increased ZPP concentrations in whole blood or erythrocytes reflect depleted iron stores and a decrease in circulating iron in the bone marrow [1,2]. Contrary to haem, ZPP and FEP fluoresce when exited at 408 nm (emission: 555 to 780 nm). Because the intensity of the fluorescent signal is proportional to the molar ratio of ZPP to haemoglobin, haematofluorometer measurements should theoretically not be influenced by the blood sample volume, pregnancy-induced haemodilution, or whether it is determined in fresh whole blood or in erythrocytes. In practice, however, measurement in washed erythrocytes can yield more valid results because washing removes haemoglobin breakdown products such as bilirubin or other serum constituents (e.g., riboflavin) that fluoresce in the same wavelength range as the porphyrins [9]. In addition to being raised in iron deficiency, ZPP can be elevated by other factors causing an inadequate supply of iron to erythroblasts (inflammation), increased erythropoiesis (haemolysis, sickle cell anaemia, thalassaemia), or disturbances in the haem synthetic pathway (lead poisoning) [1].

In many disorders, the ratio of ZPP and FEP is highly variable because of the high FEP content in reticulocytes. Using acid extraction, chelated zinc can be liberated from ZPP, yielding a larger pool of metal-free erythrocyte protoporphyrin (henceforth referred to as erythrocyte protoporphyrin, EP) [1].

We studied pregnant women with the aim to examine associations between ZPP and disorders that are common in Africa, namely *Plasmodium* infection, HIV infection, and *α*^+^-thalassaemia. In addition, we assessed the diagnostic utility of ZPP (measured in whole blood and erythrocytes) and EP, either alone or in combination with haemoglobin concentration, in detecting iron deficiency defined as plasma ferritin <15 μg/L.

## Methods

### Study population

For this study, we used samples collected at baseline for a randomised controlled trial to investigate the safety and efficacy of iron supplementation in Kenyan pregnant women. The study (www.clinicaltrials.gov: NCT01308112) received ethical clearance from review boards at the Kenyatta National Hospital/University of Nairobi, Kenya, and the London School of Hygiene and Tropical Medicine, England; written informed consent was obtained from all participating women. Field work was conducted from October 2011 to October 2012 in a rural area highly endemic for malaria in Nyanza Province, western Kenya. We set up a surveillance system to detect pregnancies in the late stage of the first trimester to the early stage of the second trimester. Pregnancy and gestational age were confirmed and determined by urine test and ultrasound examination, respectively. Immediately upon confirmation of pregnancy, women received therapeutic courses of albendazole and praziquantel against geohelminth infections and intestinal schistosomiasis, respectively.

At the second visit to the research clinic, 14 to 21 days after the initial visit, we collected a venous blood sample (6 mL) to measure haemoglobin concentrations (HemoCue301, Ängelholm, Sweden) and ZPP in whole blood and washed erythrocytes (both in duplicate; Aviv 206D, Lakewood NJ, USA). Erythrocytes were stored in DNA-stabilizing buffer (AS1, Qiagen, Valencia, CA, USA). To wash erythrocytes, blood samples were centrifuged (8 min, 600 × *g*), and plasma was removed and replaced with an equal volume of phosphate buffered saline (Medicago, Uppsala, Sweden; catalogue no. 09-2051-100). Following renewed centrifugation (8 min, 600 × *g*), the supernatant and buffy coat were discarded. For the measurement of EP, washed erythrocytes (20 μL) were transferred to 2 mL cryotubes prefilled with 0.3 mL solution 0.9% saline and 50% Celite (Sigma-Aldrich, catalogue 525235, St. Louis, MO, USA). Aliquots were stored in liquid nitrogen and dry ice until analysis for EP concentration in the Netherlands.

Plasma was stored immediately after blood collection and separation in liquid nitrogen (–196°C) in the field, and at –80°C during transport (May 2013) and subsequent storage until analysis (July 2013) in the Netherlands. Under these conditions, we believe that degradation of folate or vitamin B_12_ in stored samples was negligible.

Women were included when aged 15 to 45 years; consent had been obtained; they were likely to be available for study until 1 month after delivery and planning to deliver in the pre-designated health facility. Women were excluded when having obvious mental retardation or a metabolic disorder, a medical history of sickle cell anaemia, epilepsy, diabetes, an obstetric history suggestive of eclampsia or pre-eclampsia, were carrying multiples, the gestational age at the second visit was <13 weeks or >23 weeks, no venous blood was collected, or haemoglobin concentration was <90 g/L.

As per national guidelines, antenatal care visits should be used to provide daily supplementation with iron and folic acid, as well as intermittent preventive treatment (IPT) with sulfadoxine-pyrimethamine against malaria. However, our surveillance system captured women before they would normally make their first antenatal care visit. In our trial, we daily administered experimental supplements with or without iron but commencing only after blood collection; we did not supplement with folic acid because of a lack of published evidence of haematological benefits and because of concerns about reduced efficacy of IPT with the antifolates sulphadoxine-pyrimethamine. As part of the consent procedure, women were advised to attend regular health services to receive antenatal care as usual, including IPT and antiretroviral therapy, except that they were instructed to not take supplements with iron and/or folic acid supplied by these services or from other sources. Thus, women were unlikely to have taken supplements with iron or folic acid or at the time of blood collection, although we cannot exclude the possibility that some women had already received such supplements from shops or other sources.

### Laboratory analyses

ZPP content was measured with the AVIV ZPP haematofluorometer, Model 206D (Aviv, Lakewood Township, NJ, USA). Control samples at low, medium, and high levels (AVIV) were run after every 30 readings, while two level calibration (AVIV; low, high) samples were run twice per year. Protoporphyrins were extracted and separated from haem as described [10] and determined quantitatively using an Edinburgh Instruments FLS900 fluorescence spectrometer and a protoporphyrin IX standard (Sigma-Aldrich, catalogue 282820).

We measured plasma iron markers (concentrations of ferritin, soluble transferrin receptor, and transferrin), plasma inflammation markers (concentrations of C-reactive protein [CRP] and *α*_1_-acid glycoprotein [AGP]), vitamins (plasma concentrations of folate and total vitamin B_12_), and markers of haemolysis (plasma concentrations of lactate dehydrogenase and total bilirubin) on a Beckman Coulter UniCel DxC 880i analyser as per the manufacturer’s instructions. For test descriptions and analytic performance characteristics, we refer to the manufacturer’s website [11].

*Plasmodium* antigenaemia was assayed by dipstick tests (Access Bio Inc., Somerset, NJ, USA; CareStart, catalogue G0151 and G0171) that can detect *P. falciparum*-specific histidine-rich protein-2 (HRP2), *Plasmodium* lactate dehydrogenase (pLDH) specific to either *P. falciparum* or to non-falciparum species, i.e., *P. ovale*, *P. malariae*, or *P. vivax*. Whereas HRP2-based tests detect current or recent *P. falciparum* infection, pLDH-based tests only indicate current infection [12-14]. HIV infection was assayed using antibody tests (Alere, Waltham, MA, USA; confirmed by Unigold, Trinity Biotech, Bray, Ireland, and/or Bioline, Pantech, Umhlanga, South Africa).

We determined *α*^+^-thalassaemia genotype by polymerase chain reaction [15,16]; for practical reasons, we could perform this analysis only in the first 216 successively recruited women.

### Definitions

In our analysis of the diagnostic performance of ZPP, we defined iron deficiency as the absence or near-absence of storage iron, indicated by plasma ferritin concentration <15 μg/L [17], in women without inflammation, *Plasmodium* infection, or HIV infection. This is in accordance with the WHO’s recommendation that population iron status is measured by ferritin concentration except where inflammation is prevalent [2].

Other definitions were as follows: anaemia: haemoglobin concentration <110 g/L [18]; inflammation: plasma concentrations of CRP >10 mg/L [19] and/or AGP >1 g/L [20]; gravidity: the number of times a woman reported to have been pregnant, regardless of the outcome of these pregnancies, with twins and other multiple births counted as 1, and including the current pregnancy; *Plasmodium* infection was defined as any infection: one or more positive results for the presence of pLDH or HRP2 in plasma (dipstick tests) or *P. falciparum* DNA (PCR test); current or recent *P. falciparum* infection (similarly, but restricted to results from dipstick tests); or *P. falciparum* infection by PCR.

### Statistical analysis

Data were analysed using SPSS version 22 (IBM, Armonk, NY, USA). For ZPP, we used the mean of duplicate values; the coefficients of variation for whole blood and erythrocytes were 2.9% and 5.2%, respectively. Data were described as means (SDs), medians (25^th^ and 75^th^ percentiles), or prevalence values in the overall population or in women without inflammation (plasma concentrations CRP ≥10 mg/L or AGP ≥1.0 g/L), HIV infection, or *Plasmodium* infection.

Univariate linear regression analysis was used to explore associations between ZPP and iron markers (including anaemia), factors known or suspected to be associated with iron status (age, gestational age, gravidity, plasma concentrations of folate and total vitamin B_12_), inflammation markers, infections (*Plasmodium* infection, HIV infection), and other disorders suspected to be associated with ZPP (*α*^+^-thalassaemia, plasma concentrations of bilirubin and lactate dehydrogenase). In these analyses, ZPP values were normalised by log transformation; exponentiation of results yielded associations being expressed as percentage differences. Multivariate linear regression analysis with a backward elimination procedure was used to derive a parsimonious model of factors that were independently associated with ZPP. For whole blood ZPP and erythrocyte ZPP, this resulted in different sets of factors being included.

We assessed the diagnostic performance of ZPP (both in whole blood and erythrocytes) in detecting iron deficiency. Because plasma ferritin concentration reacts as an acute phase protein, we restricted these analyses to women without inflammation, *Plasmodium* infection, or HIV infection.

Combinations of ZPP and haemoglobin concentration may have better ability than single markers to distinguish between the presence and absence of iron deficiency. Thus, we used scatter plots and logistic discriminant analysis to assess the diagnostic performance of ZPP combined with haemoglobin concentration. Receiver operating characteristics (ROC) curves were produced using the probability of iron deficiency as a function of ZPP and haemoglobin concentration as a quantitative test outcome. Diagnostic performance was assessed by visual inspection of these curves and by assessing differences in the area-under-the-curve (AUC) with corresponding *P* values. Similar analyses were performed for EP concentration.

We subsequently assessed the diagnostic performance of ZPP as a dichotomised variable, with various thresholds. First, we used threshold values for ZPP of 70 μmol/mol haem and 40 μmol/mol haem [1,2] depending on whether the assay was conducted in whole blood or washed erythrocytes. The whole blood ZPP value of 70 μmol/mol haem [1,2,6] (2.7 μg/g haemoglobin) was derived from the 95% upper limit of the reference values for women and children participating in the US National Health and Nutrition Examination Survey II, after excluding individuals with anaemia, low transferrin saturation, and elevated blood lead concentrations. The cut-off point for erythrocyte ZPP of 40 μmol/mol haem is based on several small studies comparing iron-deficient and iron-replete individuals [9,21].

Given a diagnostic test with a binary outcome, there necessarily exists a set of paired values for sensitivity and specificity that yields a prevalence estimate that is identical to the true prevalence. Based on this premise, we determined ZPP cut-off points that would yield unbiased estimates of the prevalence of iron deficiency, with true values arbitrarily taken as 10%, 30%, and 50%. The methods to determine sensitivity and specificity pairs from the ROC curves, and thus these cut-off values for ZPP, will be described elsewhere [22].

## Results

### Population characteristics

*Plasmodium* infection was highly prevalent but with low activity, as judged by low plasma concentrations of inflammation markers, lactate dehydrogenase and bilirubin (Table 1). One-fifth of women had HIV infection and one-third had inflammation; they had poor iron status, with 37% being anaemic, 53% being iron deficient, and 27% being iron replete. Iron status was uncertain in 20% of women because they had inflammation with plasma ferritin concentrations in the normal range, which indicates either iron repletion or iron deficiency with elevated ferritin concentrations due to inflammation.

**Table 1:** **Characteristics of the populations studied**

**Characteristic**	**All women**		**Women without either inflammation,** ***Plasmodium*** **infection, or HIV infection**	
n		470		175
Age				
<20 years	20.6%	(97)	17.1%	(30)
≥20 years	79.4%	(373)	82.9%	(145)
Gestational age				
13–14 weeks	9.1%	(43)	6.3%	(11)
15–16 weeks	25.7%	(121)	25.1%	(44)
17–18 weeks	29.6%	(139)	26.3%	(46)
19–21 weeks	24.5%	(115)	30.3%	(53)
22–25 weeks	11.1%	(52)	12.0%	(21)
Gravidity				
Primigravida	18.1%	(85)	17.7%	(31)
Secundigravida	19.6%	(92)	16.0%	(28)
Multigravida	62.3%	(293)	66.3%	(116)
Plasma CRP concentration, mg/L	4.3	[2.1–10.4]	0	
Plasma AGP concentration, g/L	0.72	[0.60–0.93]	0	
Inflammation				
Plasma CRP concentration ≥10 mg/L	26.8%	(126)	0	
Plasma AGP concentration ≥1 g/L	18.1%	(85)	0	
Plasma concentrations of CRP ≥10 mg/L, or AGP ≥1.0 g/L	32.3%	(152)	0	
HIV infection	21.1%	(99)^a^	0	
*Plasmodium* infection				
Any *Plasmodium* infection,^b^ by dipstick or PCR	37.2%	(175)	0	
Current or recent *P. falciparum* infection, by either HRP2- or pLDH-based dipstick	19.5%	(91)	0	
*P. falciparum*, by PCR	34.7%	(163)	0	
Haemoglobin concentration, g/L	113.2	(11.4)	115.7	(10.8)
Anaemia (haemoglobin concentration <110 g/L)	37.2%	(175)	25.7%	(45)
Plasma ferritin concentration, μg/L	13.9	[8.2–29.2]	10.6	[7.0–18.5]
Iron status				
Iron deficient (plasma ferritin concentration <15 μg/L)	52.8%	(248)	64.6%	(113)
Iron replete (plasma ferritin concentration ≥15 μg/L, without inflammation)	27.2%	(128)	35.4%	(62)
Uncertain (plasma ferritin concentration ≥15 μg/L, with inflammation)	20.0%	(94)	0	
Whole blood ZPP, μmol/mol haem	90	[68–121]	87	[63–121]
Whole blood ZPP >70 μmol/mol haem	73.4%	(345)	69.1%	(121)
Erythrocyte ZPP, μmol/mol haem	36	[20–66]	42	[20–74]
Erythrocyte ZPP >70 μmol/mol haem	23.4%	(110)	28.6%	(50)
Erythrocyte ZPP >40 μmol/mol haem	46.4%	(218)	52.6%	(92)
EP concentration, μg/L	203	[117–428]^a^	224	[130–476]^c^
Plasma sTfR concentration, mg/L^d^	1.94	[1.48–2.63]	1.87	[1.36–2.62]
Plasma transferrin concentration, g/L	3.12	(0.56)	3.21	(0.54)
Plasma folate concentration, μg/L^e^	6.91	[5.45–9.39]^f^	6.55	[5.21–8.87]^c^
Plasma folate concentration <3 μg/L	0.6%	(3/466)^f^	0.6%	(1/174)^c^
Plasma vitamin B_12_ concentration, pmol/L^g^	425	[311–651]^f^	413	[307–638]^c^
Plasma vitamin B_12_ concentration <150 pmol/L	0.9%	(4/466)^f^	0.6%	(1/174)^c^
Plasma bilirubin concentration, μmol/L	6.9	[4.9–9.4]^f^	7.1	[4.8–9.4]^c^
*α* ^+^-thalassaemia genotype				
Normal	51.2%	[109/213]^h^	48.4%	[44]^i^
Heterozygote	41.3%	[88/213]^f^	42.9%	[39]^i^
Homozygote	7.5%	[16/213]^f^	8.8%	[8]^i^

Values indicate mean (SD), median [25^th^ and 75^th^ percentile] or % (n).

AGP, *α*_1_-acid glycoprotein protein; CRP, C-reactive protein; EP, Erythrocyte protoporphyrin; HRP2, *P. falciparum*-specific histidine-rich protein-2; pLDH, *Plasmodium*-specific lactate dehydrogenase; sTfR, Soluble transferrin receptor; ZPP:H, Zinc protoporphyrin:haem.

^a^Missing values resulted in n =468; ^b^Only one participant had infection by a *Plasmodium* species other than *P. falciparum*; ^c^Missing values resulted in n =174; Reference values ^d^0.8 1.9 mg/L and ^e^2.6 15.4 μg/L; ^f^Missing values resulted in n =466; Reference values ^g^130 700 pmol/L; Missing values resulted in ^h^n =213 and ^i^n =91.

The prevalence of iron deficiency as defined by whole blood ZPP >70 μmol/mol haem, erythrocyte ZPP >70 μmol/mol haem, and erythrocyte ZPP >40 μmol/mol haem was 73.4%, 23.4%, and 46.4%, respectively. *α*^+^-thalassaemia was common, with 41% and 8% of women being heterozygous and homozygous, respectively.

### Factors associated with ZPP

Both in univariate analysis and in parsimonious models obtained by multivariate analysis, whole blood and erythrocyte ZPP were associated with iron deficiency, anaemia, and plasma concentrations of soluble transferrin receptor (Tables 2 and 3). For example, in univariate analysis, each unit increment (1 mg/L) in plasma concentrations of soluble transferrin receptor was associated with a 32% increase in whole blood ZPP. Although whole blood ZPP seemed associated with gravidity and gestational age in multivariate analysis, such associations were not found in univariate analysis, or for erythrocyte ZPP (Table 3). Both univariate and multivariate analysis suggested that bilirubin concentration was associated with reduced erythrocyte ZPP, but such associations were not found in whole blood ZPP. *Plasmodium* infection was associated with ZPP, regardless of the case definition for *Plasmodium* infection, whether assessed in whole blood or erythrocytes, or whether examined by univariate or multivariate analysis. There was no evidence that inflammation was associated with ZPP other than that plasma *α*_1_-acid glycoprotein concentration appeared to be associated with elevated whole blood ZPP in univariate analysis. We also found no evidence that ZPP was associated with *α*^+^-thalassaemia genotype. In univariate analysis, plasma vitamin B_12_ concentration was associated with reduced ZPP, but this association disappeared in multivariate analysis. By contrast, there was no evidence for an association between plasma folate concentration and ZPP in univariate analysis, but in multivariate analysis, it was associated with increased ZPP.

**Table 2:** **Factors associated with ZPP (μmol/mol haem) measured in whole blood** ^**a**^

	**Univariate analysis**			**Multivariate analysis** ^**b**^		
	***Δ*** ^**c**^	**(95% CI)**	***P***	***Δ*** ^**c**^	**(95% CI)**	***P***
Gravidity			0.68			0.04
Primigravida	[Reference]			[Reference]		
Secundigravida	–2.3%	(–15.6% to 13.1%)		0.5%	(–9.6% to 11.8%)	
Multigravida	2.7%	(–8.9% to 15.7%)		9.4%	(0.1% to 19.6%)	
Gestational age			0.87			0.008
13–14 weeks	[Reference]			[Reference]		
15–16 weeks	5.4%	(–11.3% to 25.3%)		6.7%	(–5.6% to 20.7%)	
16–18 weeks	6.1%	(–10.4% to 25.7%)		–4.0%	(–14.9% to 8.3%)	
19–21 weeks	2.0%	(–14.3% to 21.3%)		–7.3%	(–18.2% to 5.1%)	
22–25 weeks	–1.0%	(–19.0% to 20.9%)		–11.0%	(–23.0% to –2.8%)	
Anaemia	64%	(51.2% to 77.8%)	<0.001	33.5%	(23.9% to 43.9%)	<0.001
Iron deficiency^d^	26.3%	(15.8% to 37.8%)	<0.001	16.6%	(9.1% to 24.6%)	<0.001
Plasma sTfR concentration, mg/L	31.9%	(28.1% to 35.9%)	<0.001	24.5%	(20.6% to 28.5%)	<0.001
Plasma transferrin concentration, g/L	30.9%	(21.4% to 41.2%)	<0.001	–		
Plasma folate concentration, 10 μg/L	6.1%	(–7.2% to 21.4%)	0.39	12.8%	(2.9% to 22.7%)	0.01
Plasma vitamin B_12_ concentration, 100 pmol/L	–1.8%	(–3.4% to –0.2%)	0.03	–		
Plasma total bilirubin concentration, μmol/L	0.7%	(–0.4% to 1.8%)	0.20	–		
Plasma LDH concentration, 10 IU/L	1.5%	(0.6% to 2.5%)	0.001	–		
*α* ^+^-thalassaemia genotype			0.67			
Normal	[Reference]			–		
Heterozygote	–3.8%	(–16.1% to 10.2%)		–		
Homozygote	–10.0%	(–30.2% to 16.1%)		–		
*Plasmodium* infection						
Any *Plasmodium* spp., by any dipstick or PCR	8.0%	(–1.6% to 18.4%)	0.10	–		
Current or recent *P. falciparum* infection^e^	5.1%	(–6.2% to 17.8%)	0.39	–		
*P. falciparum*, by dipstick^e^ or PCR	8.1%	(–1.4% to 18.6%)	0.10	–		
*P. falciparum*, by PCR	9.9%	(0.1% to 20.7%)	0.05	–		
HIV infection	5.2%	(–14.9% to 6.2%)	0.37	–		
Plasma CRP concentration, mg/L	0.2%	(–0.1% to 0.5%)	0.27	–		
Plasma AGP concentration, g/L	21.5%	(3.2% to 43.0%)	0.02	–		
Inflammation^f^						
Plasma CRP concentration ≥10 mg/L	5.0%	(–5.1% to 16.1%)	0.34	–		
Plasma AGP concentration ≥1.0 g/L	14.0%	(–1.5% to 27.9%)	0.03	–		
Plasma CRP concentration ≥10 mg/L, or AGP ≥1.0 g/L	6.6%	(–3.1% to 17.2%)	0.19	–		

AGP, *α*_1_-acid glycoprotein; CRP, C-reactive protein; sTfR, Soluble transferrin receptor; ZPP, Zinc protoporphyrin.

^a^ZPP values were normalised by log transformation; exponentiation of results yielded associations being expressed as percentage differences; ^b^The table shows only results for factors that were independently associated with whole blood ZPP and erythrocyte ZPP; because these final (parsimonious) models were obtained with backward elimination procedures, this resulted in different sets of factors being included for whole blood ZPP and erythrocyte ZPP; ^c^Difference; ^d^Plasma ferritin concentration <15 μg/L; ^e^Either HRP2- or pLDH-based dipstick; ^f^Plasma concentrations of CRP >10 mg/L and/or AGP >1 g/L.

**Table 3:** **Factors associated with ZPP (μmol/mol haem) measured in erythrocytes** ^**a**^

	**Univariate analysis**			**Multivariate analysis** ^**b**^		
	***Δ*** ^**c**^	**(95% CI)**	***P***	***Δ*** ^**c**^	**(95% CI)**	***P***
Gravidity			0.13			
Primigravida	[Reference]			–		
Secundigravida	23.4%	(–4.3% to 59.2%)		–		
Multigravida	23.2%	(0.0% to 51.7%)		–		
Gestational age			0.48			
13–14 weeks	[Reference]			–		
15–16 weeks	–4.6%	(–29.4% to 29.0%)		–		
16–18 weeks	13.9%	(–15.3% to 53.2%)		–		
19–21 weeks	6.1%	(–21.6% to 43.8%)		–		
22–25 weeks	–5.0%	(–33.1% to 34.8%)		–		
Anaemia	94.6%	(–67.5% to 126.2%)	<0.001	46.3%	(26.9% to 68.8%)	<0.001
Iron deficiency^d^	87.6%	(–62.1% to 117.1%)	<0.001	41.0%	(21.5% to 63.5%)	<0.001
Plasma sTfR concentration, mg/L	48.3%	(–40.0% to 57.1%)	<0.001	34.2%	(25.6% to 43.3%)	<0.001
Plasma transferrin concentration, g/L	85.3%	(–63.2% to 110.4%)	<0.001	15.6%	(0.9% to 32.5%)	0.04
Plasma folate concentration, μg/L	0.6%	(–1.8% to 2.9%)	0.64	24.0%	(3.0% to 49.3%)	0.02
Plasma vitamin B12 concentration, 100pmol/L	–3.5%	(–6.4% to –0.7%)	0.02	–		
Plasma total bilirubin concentration, μmol/L	–3.0%	(–4.8% to –1.1%)	0.002	–2.2%	(–3.7% to –0.7%)	0.005
Plasma LDH concentration, 10 IU/L	–0.4%	(–1.2% to 0.3%)	0.21	–0.1%	(–0.2% to 0.0%)	0.003
*α* ^+^-thalassaemia genotype			0.87			
Normal	[Reference]			–		
Heterozygote	–5.9%	(–25.2% to 18.3%)		–		
Homozygote	–4.7%	(–37.9% to 46.2%)		–		
*Plasmodium* infection						
Any *Plasmodium* spp., by any dipstick or PCR	–3.0%	(–17.5% to 14.1%)	0.71	–		
Current or recent *P. falciparum* infection^e^	–10.2%	(–26.3% to 9.5%)	0.29	–		
*P. falciparum*, by dipstick,^e^ or PCR	–3.0%	(–17.5% to 14.1%)	0.71	–		
*P. falciparum*, by PCR	–1.4%	(–16.3% to 16.3%)	0.87	–		
HIV infection	–8.5%	(–24.6% to 11.0%)	0.37	–		
Plasma CRP concentration, mg/L	–0.1%	(–0.6% to 0.5%)	0.82	–		
Plasma AGP concentration, g/L	1.4%	(–24.0% to 35.2%)	0.92	–		
Inflammation^f^						
Plasma CRP concentration ≥10 mg/L	–2.1%	(–18.0% to 16.8%)	0.81	–		
Plasma AGP concentration ≥1.0 g/L	3.2%	(–15.8% to 26.5%)	0.76	–		
Plasma CRP concentration ≥10 mg/L, or AGP ≥1.0 g/L	–4.2%	(–19.0% to 13.2%)	0.61	–		

AGP, *α*_1_-acid glycoprotein; CRP, C-reactive protein; sTfR, Soluble transferrin receptor; ZPP, Zinc protoporphyrin.

^a^ZPP values were normalised by log transformation; exponentiation of results yielded associations being expressed as percentage differences; ^b^The table shows only results for factors that were independently associated with whole blood ZPP and erythrocyte ZPP; because these final (parsimonious) models were obtained with backward elimination procedures, this resulted in different sets of factors being included for whole blood ZPP and erythrocyte ZPP; ^c^Difference; ^d^Plasma ferritin concentration <15 μg/L; ^e^Either HRP2- or pLDH-based dipstick; ^f^Plasma concentrations of CRP >10 mg/L and/or AGP >1 g/L.

### Diagnostic utility of ZPP

**Figure 1:**
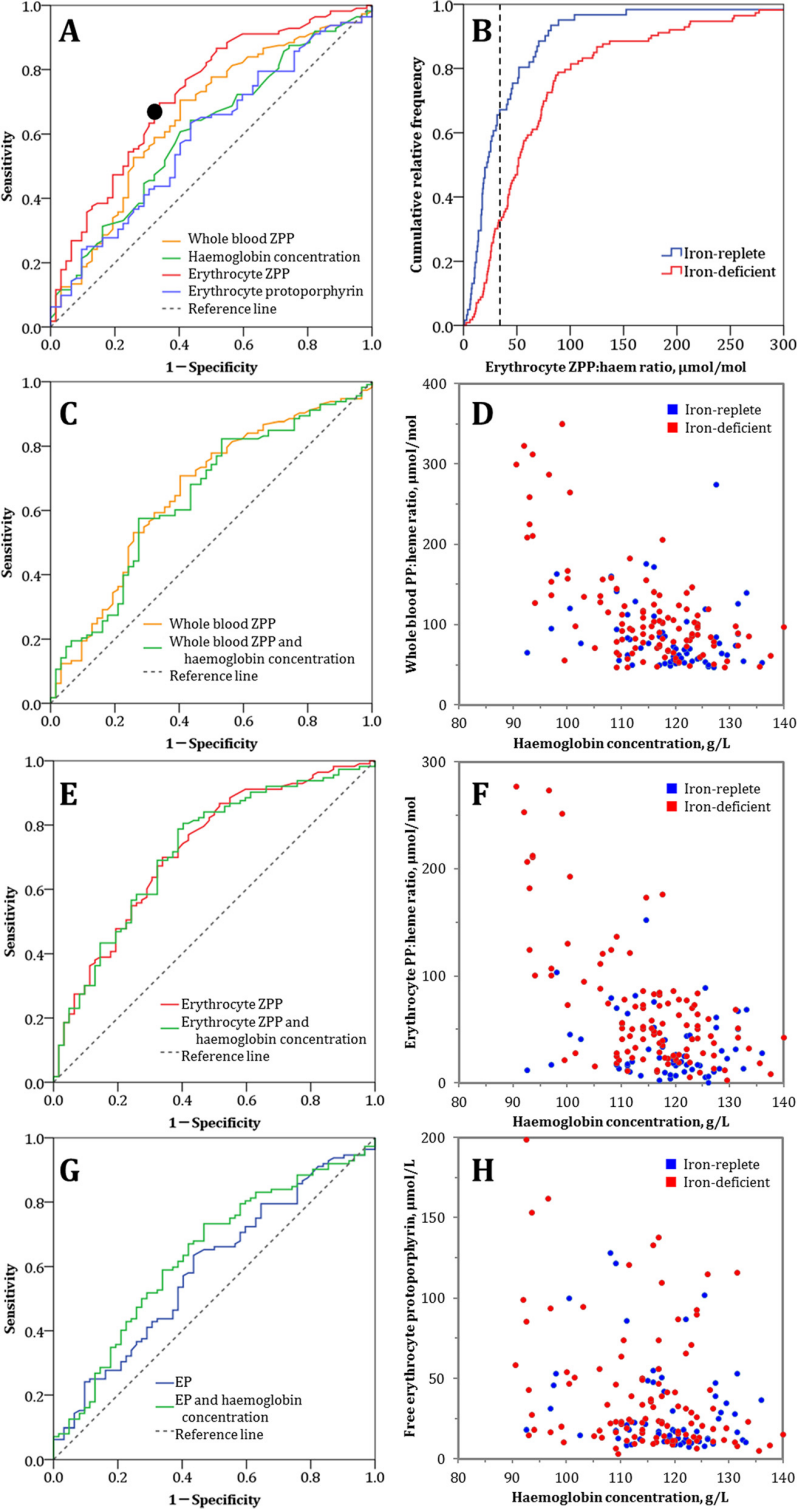
**Ability of erythrocyte protoporphyrin, either alone or combined with haemoglobin concentration, to discriminate between pregnant women with and without iron deficiency.** **(Panel A)** Receiver operating characteristics (ROC) curve for various blood markers, used alone, to discriminate between iron-deficient and iron-replete women. Area-under-the-curve (AUC; 95% CI): whole blood ZPP: (0.66; 0.57–0.74); erythrocyte ZPP: (0.73; 0.65–0.80); EP: (0.59; 0.50–0.68); haemoglobin concentration: (0.61; 0.52–0.70). **(Panel B)** Cumulative relative frequency distribution of erythrocyte ZPP, the best indicator when used as a single test **(Panel A)** to discriminate between iron-deficient and iron-replete women. The black circle in **Panel A** and the dotted black line in **Panel B** indicate the erythrocyte ZPP:haem ratio of 34 μmol/mol whereby the total diagnostic error is minimised at a prevalence of iron deficiency of 50%. **(Panels C, E, and** **G)** ROC curves for whole blood ZPP, erythrocyte ZPP, and EP, either alone or each in combination with haemoglobin concentration. AUC; 95% CI: combined whole blood ZPP with haemoglobin concentration: (0.64; 0.56–0.73); combined erythrocyte ZPP with haemoglobin concentration: (0.72; 0.64–0.80); combined EP with haemoglobin concentration: (0.64; 0.55–0.73). **(Panel D)** Bivariate scatterplot for whole blood ZPP and haemoglobin concentration, by iron status; **(Panel F)** Bivariate scatterplot for erythrocyte ZPP and haemoglobin concentration, by iron status; **(Panel H)** Bivariate scatterplot for EP and haemoglobin concentration, by iron status. Grey dashed lines in ROC curves indicate a ‘worst’ possible test, which has no discriminatory value and an area-under-the-curve (AUC) of 0.5. An ideal marker would have a curve that runs from the lower-left via the upper-left to the upper-right corner, yielding an AUC of 1.0.

In the restricted population (i.e., women without inflammation, *Plasmodium* infection, or HIV infection), whole blood ZPP, erythrocyte ZPP, and EP concentration had only modest ability to discriminate between women with and without iron deficiency (Figure 1, panels A and B). Erythrocyte ZPP scored the best out of these three markers, with an AUC of 0.73 (Figure 1, footnote). Haemoglobin concentration also performed poorly when used individually, and had no added diagnostic value when used in combination with whole blood ZPP, erythrocyte ZPP or EP concentration (Figure 1, panels C–H).

At a cut-off point of 70 μmol/mol haem, whole blood ZPP had sensitivity and specificity of 78% and 47%, respectively, of detecting iron deficiency (Table 4). This low specificity results in low positive predictive values (i.e., the probability of a test result correctly indicating iron deficiency) and gross overestimates of the prevalence of iron deficiency, particularly when the true prevalence is low. For example, at a hypothetical prevalence of 10%, the positive predictive value would be 14%, and the estimated prevalence would be 56% (Table 4).

**Table 4:** **Diagnostic performance of ZPP, measured in whole blood or erythrocytes, in detecting iron deficiency** ^**a**^ **at hypothetical prevalence values (50%, 30%, and 10%) for iron deficiency** ^**b**^

**Cut-point**	**Sensitivity**	**Specificity**	**True prevalence**	**PPV**	**NPV**	**Estimated prevalence**
**Whole blood ZPP, μmol/mol haem**						
>70^c^	78%	47%	50%	59%	68%	66%
			30%	39%	83%	61%
			10%	14%	95%	56%
>49^d^	95%	3.2%	50%	50%	39%	96%
			30%	30%	60%	96%
			10%	10%	85%	97%
>85^e^	63%	63%	50%	63%	63%	50%
>102^e^	43%	76%	30%	43%	76%	30%
>160^e^	13%	90%	10%	13%	90%	10%
**Erythrocyte ZPP, μmol/mol haem**						
>70^f^	38%	87%	50%	75%	58%	26%
			30%	56%	77%	21%
			10%	25%	93%	16%
>40^g^	64%	68%	50%	66%	65%	48%
			30%	46%	81%	42%
			10%	18%	94%	35%
>11^h^	95%	19%	50%	54%	80%	88%
			30%	34%	90%	85%
			10%	12%	97%	82%
>34^i^	67%	67%	50%	67%	67%	50%
>52^i^	48%	77%	30%	48%	77%	30%
>81^i^	27%	92%	10%	27%	92%	10%

PPV, Positive predictive value; NA, Not applicable; NPV, Negative predictive value.

^a^Defined as serum ferritin concentration <15 μg/L; ^b^Analysis restricted to women without inflammation, *Plasmodium* infection or HIV infection; ^c^Cut-off point corresponding to 2.7 μg/g, which has been selected to define the presence of iron-deficient erythropoiesis [23]; ^d^Cut-off point selected for screening, with a sensitivity of 95%; ^e^Cut-off points selected to yield unbiased estimates of the prevalence of iron deficiency (see text); ^f^Recommended range to indicate iron deficiency in the absence of infection [24]; ^g^Cut-off point recommended to distinguish between iron deficient erythropoiesis and iron sufficient erythropoiesis [2]; ^h^Cut-off point selected for screening, with a sensitivity of 95%; ^i^Cut-off points selected to yield unbiased estimates of the prevalence of iron deficiency (see text).

Erythrocyte ZPP >70 μmol/mol haem had much better specificity (87%) but a low sensitivity (38%), whilst values >40 μmol/mol haem yielded intermediate values for sensitivity and specificity (64% and 68%, respectively; values obtained from ROC curve analysis, Figure 1). With our sensitivity and specificity values, unbiased estimates for hypothetical prevalence values of 50%, 30%, or 10% would be produced at whole blood ZPP cut-off points of 85 μmol/mol haem, 102 μmol/mol haem, and 160 μmol/mol haem, respectively. Corresponding cut-off points for erythrocyte ZPP would be 34 μmol/mol haem, 52 μmol/mol haem, and 81 μmol/mol haem.

Even at a sensitivity of 95%, as may be applied for screening purposes, a negative test result obtained as whole blood ZPP ≤49 μmol/mol haem would be insufficient to rule out iron deficiency, because negative predictive values (i.e., the probability of a test result correctly indicating absence of iron deficiency), would only be 39%, 60%, and 85% at prevalence values of 50%, 30%, or 10%, respectively (Table 4).

At a prevalence of 19%, erythrocyte ZPP ≤11 μmol/mol haem (corresponding to a sensitivity of 95%) would yield 97% probability of ruling out iron deficiency, resulting in iron deficiency being excluded in 18% (=100%–82%; Table 4) of women. At higher prevalence values, this sensitivity is insufficient to rule out iron deficiency as judged by negative predictive values.

## Discussion

In the population studied, both whole blood ZPP and erythrocyte ZPP were mostly determined by iron markers including anaemia, whilst inflammation, *Plasmodium* infection, and HIV infection played only minor roles. When used individually, whole blood ZPP, erythrocyte ZPP, and EP had limited ability to discriminate between women with and without iron deficiency, whilst combining each of these markers with haemoglobin concentration had no additional diagnostic value. This limited diagnostic value was also apparent when using dichotomised variables for whole blood ZPP and erythrocyte ZPP. Conventional cut-off points for whole blood ZPP (>70 μmol/mol haem) can result in gross estimates of the prevalence of iron deficiency, particularly when the true prevalence is low. Erythrocyte ZPP may have limited value to rule out iron deficiency when used for screening in conditions with a low prevalence (e.g., 10%).

Our study was designed to investigate the diagnostic utility of ZPP in a malaria endemic, resource-poor setting that has a high prevalence of *α*^+^-thalassaemia and other haemoglobin disorders. This is particularly relevant because the World Health Organization (WHO) no longer recommends that children in malaria-endemic areas should receive universal iron supplementation; instead, children should be screened and supplementation should be restricted to those with iron deficiency [23]. Unfortunately, however, there are no simple, rapid tests available to implement this recommendation under field conditions. The WHO has pointed to the need to validate ZPP in malaria-endemic areas [23]. We believe that this lack of validity applies equally to pregnant women and children.

Iron status is commonly monitored by haemoglobin concentration, haematocrit, and plasma ferritin concentration [4]. Whereas cut-off points for these markers have been established in non-pregnant individuals, they may be unreliable in pregnancy because these markers are affected by plasma expansion. By contrast, ZPP content can be expressed as a molar ratio to haem, which should theoretically be independent of haemodilution. Thus, ZPP has been proposed as a preferred marker for iron status in pregnancy [3]. Furthermore, erythrocyte ZPP was reported to be a sensitive and specific indicator in the detection of iron deficiency in non-pregnant women and young children aged 1 to 5 years in the USA and in areas where the prevalence of elevated blood lead concentration is not high [25].

A strong point in our study was that we measured ZPP both in whole blood and in washed erythrocytes. We strictly adhered to protocol, conducted measurements in duplicate, and ran control samples as per instructions by the manufacturer. Lead exposure in our study population was probably very low. We ensured comprehensive assessment of iron status in all the participants using different markers as recommended by various guidelines [2,26]. The iron markers, inflammation markers, and haemolysis markers reported in this study, as well as EP concentrations, were assessed independently by laboratories that were not involved in the fieldwork. In addition, by using a broad array of methods (dipsticks, PCR, and histopathology), we probably detected most asymptomatic *Plasmodium* infections. The high acceptance of HIV testing (98%) enabled us to study the diagnostic performance of ZPP in the presence of HIV as a chronic infection.

We found no strong, consistent evidence that indicators of haemolysis (bilirubin, lactate dehydrogenase), infection (*Plasmodium*, HIV), or inflammation were associated with ZPP. An explanation may be that our study subjects probably had high levels of acquired protective immunity against malaria, whilst effects of HIV infection may have been suppressed by the use of antiretroviral therapy.

Although fewer than 1% of women had low plasma concentrations of folate (<3 μg/L) and vitamin B12 (<150 pmol/L), these findings should be interpreted with caution, because these plasma markers reflect both intake and stores, and are subject to large inter-laboratory and inter-assay variability [27].

When analysing the diagnostic performance of ZPP, we based our definition of iron deficiency on plasma ferritin concentration, whilst restricting our dataset to women without inflammation, *Plasmodium* infection, or HIV infection. Plasma ferritin concentration reacts as an acute phase protein and can be elevated in the presence of infection-induced inflammation independently of iron status. It can be argued that, as an alternative, the ratio of concentrations of soluble transferrin receptor/log ferritin can be used in an unrestricted dataset. This ratio, however, is a marker of body iron content and we found its use to define iron deficiency problematic: it will be influenced by inflammation because serum ferritin concentration is one component of the ratio [2]. It will also be influenced by *Plasmodium* infection independent of iron status, since serum transferrin receptor concentrations also reflect increased erythropoiesis induced by *Plasmodium* infection [28,29]. Additional arguments against the use of this ratio in this paper are provided in its pre-publication history (available online).

Plasma ferritin concentration with an adjusted cut-off value (e.g., 30 μg/L) has been advocated to define iron deficiency in the presence of inflammation. Implicitly, however, this definition assumes that the effect of inflammation on plasma ferritin concentration is identical for all individuals within a population or across populations, whereas in reality, this will depend on many factors including the degree and duration of inflammation, as well as immunity and its proxy indicators (e.g., age). Particularly in a population with *Plasmodium* infection, there is currently no marker or combination of markers to accurately diagnose iron deficiency.

Our study had several limitations. First, for practical reasons, we could not assess the *α*^+^-thalassaemia status of all participants. However, we found no associations between ZPP and *α*^+^-thalassaemia in the women in whom *α*^+^-thalassaemia genotype was established. Secondly, we studied pregnant women. Although we do not expect that the diagnostic performance of ZPP is better in children, we cannot exclude this possibility. In addition, other conditions and genetic disorders such as sickle cell anaemia and G6PD, which may influence the diagnostic utility of ZPP, were not studied.

Several other studies also found that detection of iron deficiency by ZPP leads to marked overestimates of the prevalence of iron deficiency [6,7,30,31]. For example, in Kenyan children, this prevalence was 80% for ZPP >80 μmol/mol haem versus 41% for ferritin concentration <12 μg/L (after correction for inflammation) [6]. In Tanzanian children without *Plasmodium* infection, corresponding values were 56% versus 40% [7]. These discrepancies may have been due at least in part to inflammation or infection [2,24]. Several studies have shown that ZPP content in whole blood can be markedly higher than values measured in washed erythrocytes. Various reasons, including interference by bilirubin, have been cited [8,20,32-34]. Our findings show, however, that this overestimation is also in large part due to low specificity to ZPP at conventionally used cut-off points, whether measured in whole blood or in erythrocytes. Selection of cut-points for dichotomized diagnostic tests should depend on the diagnostic aims. When used as an initial screening marker to manage iron deficiency, ZPP should be highly sensitive, with a view to rule out iron deficiency (i.e., a high negative predictive value, no longer needing work-up) or to identify individuals who are iron deficient or who need further diagnostic work-up. Our findings show, however, that a high sensitivity will inevitably be accompanied by a low specificity, and thus an unacceptably low negative predictive value. For example, in Table 4, we have shown that for whole blood ZPP, a sensitivity of 95% can be obtained with a cut-off point of 49 μmol/mol haem. However, the corresponding specificity is 3.5%, resulting in negative predictive values that will be unacceptably low with true prevalence values for iron deficiency in most conditions. As an exception, erythrocyte ZPP may have limited value to rule out iron deficiency in populations with low prevalence of iron deficiency (Table 4). These results were obtained in a restricted dataset, with exclusion of women with inflammation, *Plasmodium* infection, or HIV infection. The diagnostic performance of ZPP in an unrestricted dataset would presumably have been even worse.

When applied to estimate the prevalence of iron deficiency, ZPP cut-off points can be calibrated to produce estimates that are not biased by diagnostic error.

## Conclusions

In this population, both whole blood ZPP and erythrocyte ZPP have little diagnostic utility as a screening marker to manage iron deficiency, whether used as single tests or combined with haemoglobin concentration. When used to estimate the prevalence of iron deficiency, conventional cut-off points for whole blood ZPP can result in marked overestimates. Based on these findings, guidelines on the use of ZPP to assess iron status in individuals or populations of pregnant women need review.

## Abbreviations

AGP: *α*_1_-acid glycoprotein
AUC: Area-under-the-curve
CRP: C-reactive protein
EP: Erythrocyte protoporphyrin
FEP: Free erythrocyte protoporphyrin
HRP2: Histidine-rich protein-2
IPT: Intermittent preventive treatment
pLDH: *Plasmodium* lactate dehydrogenase
ROC: Receiver operating characteristics
WHO: World health organization
ZPP: Zinc protoporphyrin

## References

[1] NCCLS (1996). *Erythrocyte Protoporphyrin Testing; Approved Guideline*. NCCLS document C42-A.

[2] WHO/CDC (2007). Assessing the Iron Status of Populations.

[3] Schifman RB, Thomasson JE, Evers JM (1987). Red blood cell zinc protoporphyrin testing for iron deficiency anemia in pregnancy. Am J Obstet Gynecol.

[4] Romslo I, Haram K, Sagen N, Augensen K (1983). Iron requirement in normal pregnancy as assessed by serum ferritin, serum transferrin saturation and erythrocyte protoporphyrin determinations. Br J Obstet Gynaecol.

[5] Harthoorn-Lasthuizen EJ, Lindemans J, Langenhuijsen MMAC (2000). Erythrocyte zinc protoporphyrin testing in pregnancy. Acta Obstet Gynecol Scand.

[6] UN Children’s Fund/UN University/World Health Organization (2001). Iron Deficiency Anaemia: Assessment, Prevention, and Control. A Guide for Programme Managers.

[7] Grant FKE, Martorell R, Flores-Ayala R, Cole CR, Ruth LJ, Ramakrishnan U, Suchdev PS (2012). Comparison of indicators of iron deficiency in Kenyan children. Am J Clin Nutr.

[8] Stoltzfus RJ, Chwaya HM, Albonico M, Schulze KJ, Savioli L, Tielsch JM (1997). Serum ferritin, erythrocyte protoporphyrin and hemoglobin are valid indicators of iron status of school children in a malaria-holoendemic population. J Nutr.

[9] Hastka J, Lasserre J, Schwarzbeck A, Strauch M, Hehlmann R (1992). Washing erythrocytes to remove interferents in measurements of zinc protoporphyrin by front-face hematofluorometry. Clin Chem.

[10] Piomelli S (1977). Free erythrocyte porphyrins in the detection of undue absorption of Pb and of Fe deficiency. Clin Chem.

[11] *Beckman Coulter.* [https://www.beckmancoulter.com/]

[12] Makler MT, Piper RC, Milhous WK (1998). Lactate dehydrogenase and the diagnosis of malaria. Parasitol Today.

[13] Piper R, Lebras J, Wentworth L, Hunt-Cooke A, Houzé S, Chiodini P, Makler M (1999). Immunocapture diagnostic assays for malaria using *Plasmodium* lactate dehydrogenase (pLDH). Am J Trop Med Hyg.

[14] Moody A (2002). Rapid diagnostic tests for malaria parasites. Clin Microbiol Rev.

[15] Veenemans J, Andang’o PEA, Mbugi EV, Kraaijenhagen RJ, Mwaniki DL, Mockenhaupt FP, Roewer S, Olomi RM, Shao JF, van der Meer JWM, Savelkoul HFJ, Verhoef H (2008). α^+^-Thalassemia protects against anemia associated with asymptomatic malaria: evidence from community-based surveys in Tanzania and Kenya. J Infect Dis.

[16] Veenemans J, Jansen EJS, Baidjoe AY, Mbugi EV, Demir AY, Kraaijenhagen RJ, Savelkoul HFJ, Verhoef H (2011). Effect of α(+)-thalassaemia on episodes of fever due to malaria and other causes: a community-based cohort study in Tanzania. Malar J.

[17] WHO (2011). Serum Ferritin Concentrations for the Assessment of Iron Status and Iron Deficiency in Populations.

[18] WHO (2011). Haemoglobin Concentrations for the Diagnosis of Anaemia and Assessment of Severity.

[19] Nielsen FR, Bek KM, Rasmussen PE, Qvist I, Tobiassen M (1990). C-reactive protein during normal pregnancy. Eur J Obstet Gynecol Reprod Biol.

[20] Filteau S, Morris S, Abbott R, Tomkins A, Kirkwood B, Arthur P, Ross D, Gyapong J, Raynes J (1993). Influence of morbidity on serum retinol of children in a community-based study in northern Ghana. Am J Clin Nutr.

[21] Hastka J, Lasserre J, Schwarzbeck A, Hehlmann R (1994). Central role of zinc protoporphyrin in staging iron deficiency. Clin Chem.

[22] Talsma EF, Verhoef H, Brouwer ID, Mburu-de Wagt AS, Hulshof PJM , Melse-Boonstra A: **Proxy markers of serum retinol concentration, used alone and in combination, to assess population vitamin A status in Kenyan children: a cross-sectional study.** *BMC Med* [in press]. 10.1186/s12916-014-0256-5 PMC4324407 25856672

[23] Senga EL, Koshy G, Brabin BJ (2012). Zinc erythrocyte protoporphyrin as marker of malaria risk in pregnancy - a retrospective cross-sectional and longitudinal study. Malaria J.

[24] WHO (2007). Conclusions and recommendations of the WHO Consultation on prevention and control of iron deficiency in infants and young children in malaria-endemic areas. Food Nutr Bull.

[25] Mei Z, Parvanta I, Cogswell ME, Gunter EW, Grummer-Strawn LM (2003). Erythrocyte protoporphyrin or hemoglobin: which is a better screening test for iron deficiency in children and women?. Am J Clin Nutr.

[26] Pavord S, Myers B, Robinson S, Allard S, Strong J, Oppenheimer C: *UK Guidelines on the Management of Iron Deficiency in Pregnancy.* London, UK: British Committee for Standards in Haematology; 2011. [http://www.bcshguidelines.com/documents/UK_Guidelines_iron_deficiency_in_pregnancy.pdf] Accessed 15 July 2014. 10.1111/j.1365-2141.2011.09012.x 22512001

[27] FNB/IOM (1998). Dietary Reference Intakes for Thiamin, Riboflavin, Niacin, Vitamin B6, Folate, Vitamin B12, Pantothenic Acid, Biotin, and Choline.

[28] Verhoef H, West CE, Ndeto P, Burema J, Beguin Y, Kok FJ (2001). Serum transferrin receptor concentration indicates increased erythropoiesis in Kenyan children with asymptomatic malaria. Am J Clin Nutr.

[29] Verhoef H, West CE, Kraaijenhagen R, Nzyuko SM, King R, Mbandi MM, van Laatum S, Hogervorst R, Schep C, Kok FJ (2002). Malarial anemia leads to adequately increased erythropoiesis in asymptomatic Kenyan children. Blood.

[30] Asobayire FS, Adou P, Davidsson L, Cook JD, Hurrell RF (2001). Prevalence of iron deficiency with and without concurrent anemia in population groups with high prevalences of malaria and other infections: a study in Côte d’Ivoire. Am J Clin Nutr.

[31] Crowell R, Ferris AM, Wood RJ, Joyce P, Slivka H (2006). Comparative effectiveness of zinc protoporphyrin and hemoglobin concentrations in identifying iron deficiency in a group of low-income, preschool-aged children: practical implications of recent illness. Pediatrics.

[32] Schifman RB, Finley PR (1981). Measurement of near-normal concentrations of erythrocyte protoporphyrin with the hematofluorometer: influence of plasma on “front-surface illumination” assay. Clin Chem.

[33] Janousek SJ, Rosa L, Jirova D, Kejlova K (2010). Oxidative stress may modify zinc protoporphyrin/heme ratio in hematofluorometry. Int J Lab Hematol.

[34] Buhrmann E, Mentzer WC, Lubin BH (1978). The influence of plasma bilirubin on zinc protoporphyrin measurement by a hematofluorimeter. J Lab Clin Med.

